# Modelling decision-making biases

**DOI:** 10.3389/fncom.2023.1222924

**Published:** 2023-10-20

**Authors:** Ettore Cerracchio, Steven Miletić, Birte U. Forstmann

**Affiliations:** Department of Psychology, University of Amsterdam, Amsterdam, Netherlands

**Keywords:** cognitive modelling, decision-making bias, attention, prior probability, DDM, SDT, EAM

## Abstract

Biases are a fundamental aspect of everyday life decision-making. A variety of modelling approaches have been suggested to capture decision-making biases. Statistical models are a means to describe the data, but the results are usually interpreted according to a verbal theory. This can lead to an ambiguous interpretation of the data. Mathematical cognitive models of decision-making outline the structure of the decision process with formal assumptions, providing advantages in terms of prediction, simulation, and interpretability compared to statistical models. We compare studies that used both signal detection theory and evidence accumulation models as models of decision-making biases, concluding that the latter provides a more comprehensive account of the decision-making phenomena by including response time behavior. We conclude by reviewing recent studies investigating attention and expectation biases with evidence accumulation models. Previous findings, reporting an exclusive influence of attention on the speed of evidence accumulation and prior probability on starting point, are challenged by novel results suggesting an additional effect of attention on non-decision time and prior probability on drift rate.

## 1. Introduction

In a decision-making task, a response bias can occur when one choice is preferred over its alternative or actively avoided (Leite and Ratcliff, 2011). Conversely, an unbiased decision involves equal competition between all possible choices (Dricu et al., 2017). Unbiased decisions, no matter how theoretical, serve as a benchmark to detect response biases and control for undesired factors. For example, researchers often compare conditions of biased attention with neutral ones. Attention and expectation are two common experimental manipulations that can induce a response bias in decision-making tasks.

A controversy exists about the exact definition of attention and addressing this issue is beyond the scope of this article. Here, we define attention as “the flexible control of limited computational resources” (Lindsay, 2020). Several attentional mechanisms are experimentally manipulated in perceptual decision-making tasks. Spatial attention allows us to prioritize information processing at a given location, like covertly attending to one side of the screen (Carrasco, 2018). Temporal attention is the ability to focus in time (Ramirez et al., 2021), and, in a similar way, feature-based and object-based attention prioritize the processing of relevant features (White and Carrasco, 2011) or objects in the environment (Ciaramitaro et al., 2011). Response biases between conditions have been detected for spatial (Ghaderi-Kangavari et al., 2023), temporal (Denison et al., 2017), feature-based (Ho et al., 2012), and object-based attention (Ciaramitaro et al., 2011).

Similarly to attention, tasks manipulating the subjects’ expectation, i.e., prior knowledge about the choices’ probability, can induce response biases in the behavioral data (Mulder et al., 2012). We usually refer to this contextual information as prior probability. In decision-making tasks, instructions informing the participant about the stimulus’ prior probability are often used to induce perceptual expectations (Cheadle et al., 2015). On a neural level, Kok et al. (2014, 2017) demonstrated in a series of functional magnetic resonance imaging (fMRI) and magnetoencephalography (MEG) experiments that perceptual predictions evoked similar BOLD activity patterns to those evoked by the stimuli in the primary visual cortex. One appealing idea is that the actual sensory input is compared to the prediction template to evaluate the quality of the prediction. If the input mismatches the prediction, a prediction error signal is generated (Smout et al., 2019).

Response biases may also be induced by reward manipulations (Mulder et al., 2012); we refer to this as a *reward bias*. Another bias is the *choice history bias*, the replication of the previous answer(s) to guide the current decision (Urai et al., 2019). Moreover, consistency in the direction of evidence (e.g., the left stimulus is consistently brighter than the right stimulus) has also been shown to bias accuracy, confidence and response times (Glickman et al., 2022). This is known as *consistency bias*. In sum, the term *decision-making bias* encompasses response biases induced by various manipulations.

### 1.1. Aim

The aim of this paper is to provide researchers with a conceptual overview of both the theoretical principles and empirical applications related to decision-making biases. More specifically, we will focus on the modelling approaches researchers use to study attention and expectation. We start by describing statistical models and then move to cognitive models of decision-making (Townsend and Ashby, 1983). Here we will describe their advantages over statistical models. We review studies that used both Signal Detection Theory (SDT; Green and Swets, 1966) and the Diffusion Decision Model (DDM; Ratcliff, 1978), as they stand out as well-established cognitive models (a thorough description of SDT and DDM is present in Section 2.3). We also compare these two cognitive models and argue that the latter offers greater insight into the data due to the analysis of response times (RT) distributions.

With respect to the latter models, response biases are particularly suitable for cognitive modelling because they provide valuable information about the underlying cognitive processes that lead to the observed behavior. This is because response biases can help identify the specific cognitive mechanisms involved in decision-making. For example, a response bias favoring one choice option over another may indicate that the participant is relying more heavily on certain perceptual cues or using a specific strategy. This information can then be incorporated into cognitive models to better understand how these cognitive mechanisms operate. Moreover, it is possible to jointly model both behavioral and neural activity measures (Turner et al., 2017). This simultaneous modelling offers the possibility of gaining a neural understanding of these cognitive processes. Response biases can also be used to test and compare different cognitive models. By comparing the fit of different models to the observed data, researchers can identify the model that best describes the putative underlying cognitive mechanisms. Lastly, response biases can be used to study individual differences in decision-making. By examining how response biases vary across different individuals or populations, researchers can identify factors that influence decision-making and tailor interventions accordingly.

Overall, response biases are a valuable tool for cognitive modelling as they provide rich information about the underlying cognitive processes that drive behavior, allow for the comparison of different cognitive models, and facilitate the study of individual differences in decision-making.

## 2. Modelling biases

### 2.1. Verbal and formal theories of decision-making

Researchers studying response biases are usually interested in testing a specific theory of decision-making. Nonetheless, theories of decision-making vary in their form. On the one hand, *formal theories* are characterized by mathematical tools and formal concepts (van Rooij and Blokpoel, 2020). On the other hand, *verbal theories* are verbally expressible intuitions (van Rooij and Blokpoel, 2020).

Verbal and formal theories are the two sides of the theoretical modelling “coin.” Intuitions about psychological phenomena need to be formally transcribed to be quantitatively tested. Similarly, the formalization process of a verbal theory can highlight practical limitations that were not previously conceptualized. The resolution of these practical limitations results in a theory that quantitatively predicts data. In this way, the process of creating a theory is characterized by the cyclical alternation between verbal and formal theories.

Nonetheless, researchers do not test formal theories solely. Contrarily, it is common to encounter studies that aim at testing a verbal theory. Testing verbal and formal theories has different implications that affect the whole empirical process, from hypotheses, to predictions, testing, and inferences (see Table 1).

**Table 1:** Comparison of verbal and formal theories of decision-making (DVs: dependent variables).

Theory	Approach	Analysis	Prediction	Testing	Inference
Verbal	Hypothesis-based	Based on DVs	Qualitative	Statistical testing	Effect of manipulation on DVs
Formal	Model-based	Based on model’s parameters	Quantitative	Model fitting	Effect of manipulation on model parameters

### 2.2. Statistical models

Verbal theories can be, and usually are, tested and validated via statistical models. Statistical models do not make assumptions about the underlying cognitive processes that result in decision-making, but rather focus on describing the patterns observed in the data. The description and decomposition of variance in the data can in turn inform whether the data are in line with the theory and drive the further formalization of the verbal theory under investigation.

A substantial set of studies that probed decision-making biases applied statistical models to analyze their data (Hsu et al., 2018; Huang et al., 2018; Jigo et al., 2018; Garcia-Lazaro et al., 2019; Kveraga et al., 2019; Smout et al., 2019, 2020; Aitken et al., 2020; Moerel et al., 2022). An example is the event-related potential (ERP) study proposed by Doherty et al. (2005) which jointly investigated the effect of temporal and spatial expectation on decision-making. The task consists of a red ball moving in steps from the left to the right side of the screen. The ball is then occluded by a grey vertical bar for two steps. When it reappears on the other side of the occluded area, participants are asked to respond in case a black dot was present inside the ball. A 2-by-2 design produced a total of four conditions, namely temporal and spatial expectation (ST), spatial expectation only (S), temporal expectation only (*T*) and neither of the two (*N*). The four conditions were set up using the movement of the ball. The ball moved either with or without a constant spatial trajectory to manipulate spatial expectation and, with or without a constant step duration to manipulate temporal expectation. The authors analyzed the behavioral data using a repeated-measures two-way ANOVA (and *post hoc* paired-samples *t* tests), which examines the influence of two categorical independent factors (temporal and spatial expectations) on a continuous dependent variable (response time). Provided that the assumptions of the parametric estimation of *p*-values corresponding to the expectation manipulations have been met [which can be challenging, given the non-Gaussian nature of RT distributions and repeated measures paradigms (Girden, 1992)], these models can give valuable insights in the presence and size of biases caused by experimental manipulations. The authors reported faster response times in the *S* and *T* conditions compared to the *N* condition, but the interaction between the two factors was not significant. The reduction in mean response time in the ST condition was larger than the effects in the *S* and *T* conditions alone, indicating a so-called additive effect.

In sum, the authors provided evidence using a statistical model for an enhancing effect of both spatial and temporal expectation on response speed, plus an additive effect when both expectations were induced. Nonetheless, the verbal theory used for hypothesis generation cannot quantify the relative influences of these biases in the ST condition nor identify which cognitive processes (e.g., evidence accumulation, criterion or motor response time) have induced such changes. Thus, one may argue that the interpretation of these results remains an approximation while, next to statistical models, mathematical cognitive models may overcome this limitation. By specifying formal assumption about the decision process and subsequently testing which model best describes the data, mathematical cognitive models offer the possibility of decomposing behavioral data into a more structured set of cognitive processes. For the very same reason, mathematical cognitive models cannot account for any underlying cognitive factor that is not specified in the model *a priori*.

### 2.3. Cognitive models

Mathematical cognitive models permit the decomposition of the observed performance into isolated contributions of relevant cognitive processes (Heathcote et al., 2015; Forstmann et al., 2016). Nonetheless, the relationship between statistical and cognitive models is not competitive; instead, it can be understood as a hierarchy where statistical models offer preliminary inference and cognitive models delve deeper into cognitive processes by mathematically formalizing their assumptions. The fit of the model gives insights in how well the cognitive process model can capture the empirical data. Thus, cognitive models yield falsifiable, transparent, and reproducible descriptions of the cognitive processes inferred by the behavioral data (Frischkorn and Schubert, 2018).

#### 2.3.1. Signal detection theory

**Figure 1:**
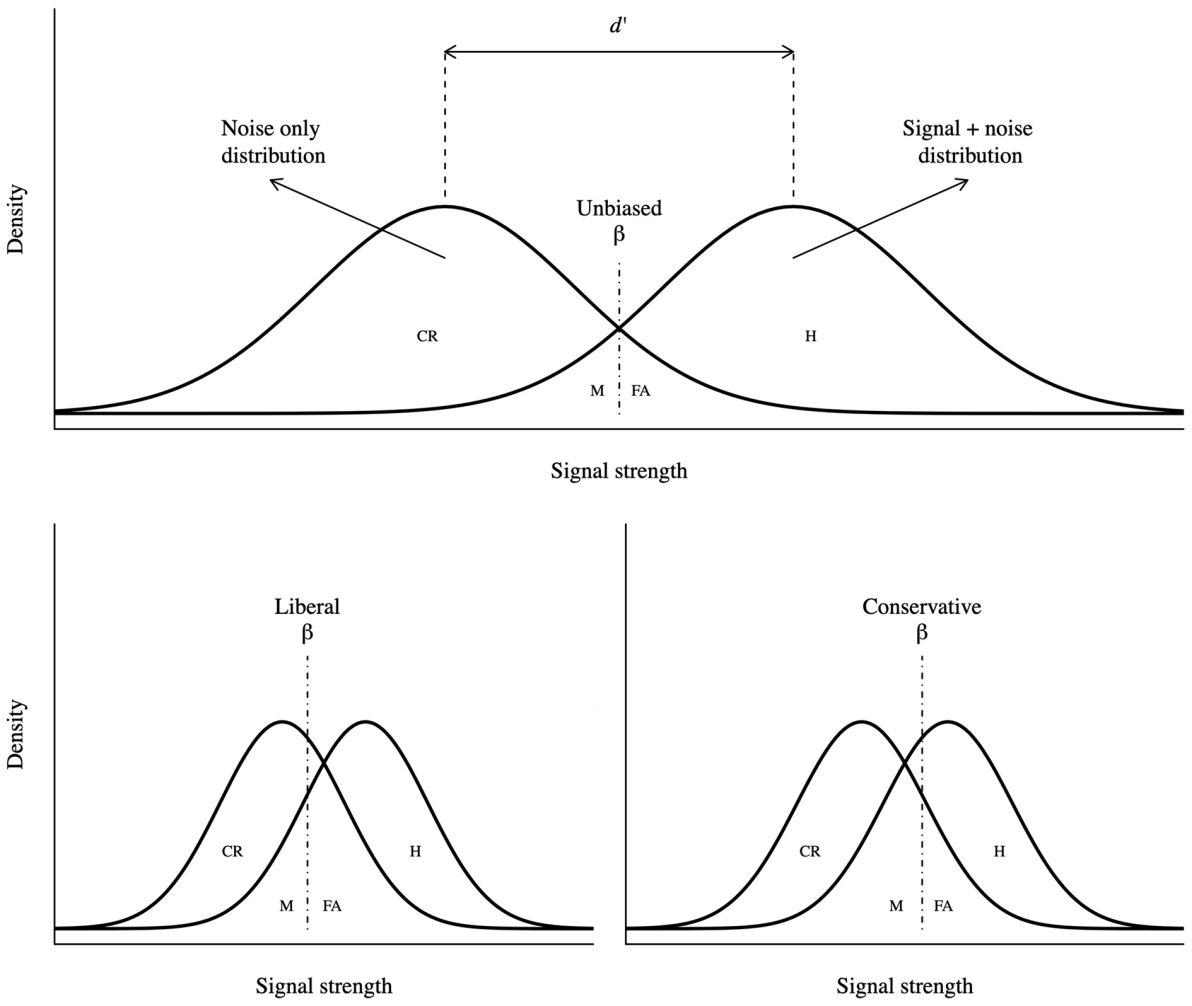
Visual representation of SDT. Sensitivity is reported as d’, response bias as beta, hits as H, false alarms as FA, misses as M and correct rejections as CR. A more liberal criterion is characterized by more positive responses, resulting in higher hit and false alarm rates. Parallelly, a more conservative criterion is characterized by more negative responses, resulting in higher miss and correct rejection rates.

Signal detection theory is a widely used framework to study decision-making and perception. In a simple psychophysics experiment, the observer gathers noisy evidence (e) from the environment and has two categorical options, stimulus is present (h1), and stimulus is not present (h2; see Figure 1). This theory is a means to infer which of the hypotheses caused the evidence (Gold and Shadlen, 2007). If the observer correctly reports the presence or absence of the stimulus, the trial is labelled Hit (*H*) and Correct rejection (CR), respectively. Conversely, if the observer incorrectly reports the stimulus presence or absence, the trials are labelled as False alarm (FA) and Miss (M), respectively. In a two-choice task, the ratio of the two likelihoods (h1 and h2) defines the *decision variable* as follows:


$$L_{12}\left(e\right)=P\left({e|h}_{1}\right)/P\left({e|h}_{2}\right)$$

The likelihood ratio (β) is one measure of *criterion*, also called response bias, which acts as a threshold with respect to the decision variable:


$$L12\left(e\right)\geq \beta$$

Thus, the criterion determines whether the participant systematically responds more positively or negatively (Vickers, 1979; Macmillan and Creelman, 2005); SDT effectively defines and provides a formal description of a *bias*. As illustrated in Figure 1, a more liberal criterion implies higher Hit and False alarm rates, while a more conservative criterion implies higher Miss and Correct rejection rates, compared to an unbiased criterion.

Conversely, the *sensitivity* measures the ability of discrimination, which quantifies accurate performance (Vickers, 1979; Macmillan and Creelman, 2005). Conceptually, it represents the distance between the “noise only” and “noise + signal” distributions (see Figure 1). The most common measure of sensitivity (Green and Swets, 1966) is d’, which is formalized in terms of z as follows:


$$d'\;=\;z\left(H\right)-z\left(FA\right)$$

The same sensitivity value can be obtained from different combinations of FA and H proportions. This equivalence in sensitivity is captured by the so-called receiver operating characteristic (ROC) curve, which describes the sensitivity of any classifier. The criterion, desirably independent of sensitivity, captures the remaining component of the parameter space. In reality, only one measure of criterion – the criterion location (c) – is statistically independent from sensitivity (Macmillan and Creelman, 2005). Such independence is also depicted by the ROC curve, as a shift in criterion does not alter the curve itself but is represented as different points along the same ROC curve (meaning different combinations of FA and H proportion).

The seemingly simplicity of SDT may explain the wide use of this framework as a cognitive model. There are several studies that have used SDT to investigate prior probability bias, but the results depict an inconsistent picture. Prior probability manipulations have been found to mainly influence criterion (de Lange et al., 2013; Cheadle et al., 2015; Kaneko and Sakai, 2015) but also sensitivity (Stein and Peelen, 2015). Conversely, substantial work using the attentional cue paradigm converged on the idea that spatial attention has an enhancing effect on discriminability, which is captured by the sensitivity (d’) parameter (Bashinski and Bacharach, 1980; Downing, 1988; Hawkins et al., 1990; Cheadle et al., 2015).

**Figure 2:**
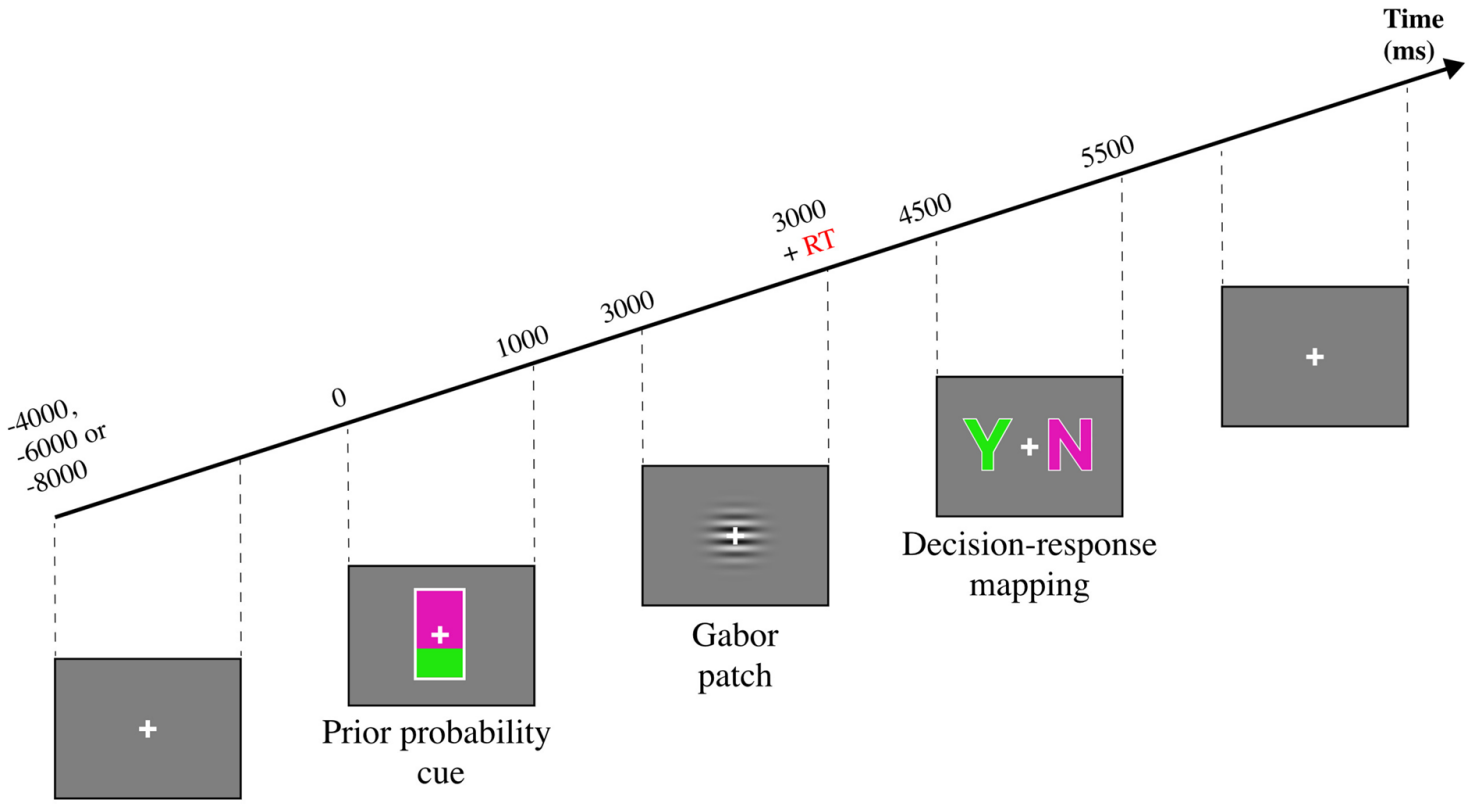
Experimental task from Kaneko and Sakai (2015). A mostly green probability cue indicates a 67% probability of seeing a moving Gabor patch, while a mostly purple cue indicates a 33% probability of seeing a moving Gabor patch. The Gabor patch is shown until the participant presses a key, indicating that he has decided. A subsequent decision-response mapping cue instructs the participants to report whether the Gabor patch was moving or not.

The study of Kaneko and Sakai (2015) is of relevance for this review because it highlights the benefits of applying cognitive modelling to behavioral data. In this study, the authors investigated the differences between prior probability and choice history bias with a perceptual decision-making task (see Figure 2), as it is unclear whether these biases arise via the same mechanism. The aim of the task was to report whether the target Gabor patch was moving [Target (+)] or not [Target (−)]. Prior probability was manipulated via a probabilistic cue preceding the Gabor patch. Two cues indicated whether the Gabor patch was likely (67%) or unlikely (33%) to move. The authors conducted a multilevel logistic regression analysis (a statistical model) on the behavioral data and reported that the previous decision, the cue, and the target were the factors that mostly contributed to target detection. Response times were enhanced by valid cues and impaired by invalid cues, whereas the previous decision did not affect RT. Moreover, the best-fitting model reported a significant interaction between previous decision and target on accuracy, which led the authors to suggest an effect of choice history bias only when the Gabor patch was moving. Conversely, cue and previous decision did not interact, suggesting distinct mechanisms biasing the decision process. The authors also modelled the dataset with SDT to determine the effects of prior probability and choice history on the model’s parameters. The authors report a main effect of choice history on sensitivity and a main effect of prior probability on criterion. This result further corroborates a view in which these manipulations affect decision-making via distinct mechanisms.

The main difference between the two analyses conducted by the authors is the extent to which they can link results to specific *factors* influencing the decision process. Even if the results of the statistical model converged with the SDT ones, only the latter ones can be associated with latent factors which are cognitively significant. The statistical model’s parameters have not been designed to map onto latent cognitive processes. Thus, while the statistical model’s parameters describe unspecified factors, the SDT’s parameters are directly linked with latent cognitive processes.

#### 2.3.2. Evidence accumulation models

While SDT explains decision-making based on a single observation of evidence, the Sequential Probability Ratio Test (SPRT; Wald, 1945) introduces the idea of making decisions through a sequential evidence accumulation process. In SPRT, the log likelihood ratio between two competing hypotheses is updated after every observation. In turn, evidence accumulation models (EAMs) build upon this framework to better describe the whole decision-making process, including sensory encoding and motor execution time. Thus, SDT and EAMs present themselves as static and dynamic variants within the same family of hypothesis tests (Griffith et al., 2021). By estimating the accumulation of evidence over time, EAMs not only predict accuracy but also response time distributions – which have been shown to be crucial when linking behavioral and neural data (Gold and Shadlen, 2007). The assumptions shared between EAMs constitute a formal theory of decision-making (Evans and Wagenmakers, 2020) with three main assumptions: (1) evidence supporting each choice is accumulated over time, (2) the accumulation is characterized by random fluctuations, and (3) the decision is taken when enough evidence favoring one choice has been accumulated up to a threshold (Bogacz et al., 2006). While these assumptions generally hold for the whole class of EAMs, each model has different characteristics, parameters, and assumptions. For example, a major distinction between evidence accumulation models pertains to the number of accumulators. The DDM has a single accumulator with two boundaries, thus describing a two-alternative forced choice. Race models, such as the Racing Diffusion Model (RDM; Tillman et al., 2020), the Linear Ballistic Accumulator (LBA; Brown and Heathcote, 2008) and the Leaky Competing Accumulator (LCA; Usher and McClelland, 2001), can account for multiple responses and can thus be applied to a wider range of tasks. For a detailed comparison between EAMs see Ratcliff et al. (2016).

In the past, both the RDM (Tillman et al., 2020) and the LBA (Forstmann et al., 2010; Nishiguchi et al., 2019) have been used to investigate decision-making biases. Nonetheless, the DDM remains the most used EAM in the response bias research (Mulder et al., 2012; Nunez et al., 2017; Garton et al., 2019; Klatt et al., 2020; Ghaderi-Kangavari et al., 2022, 2023) and it has been shown to be the optimal decision-making mechanism for two-alternative forced choice tasks (Bogacz et al., 2006). Because of its extended applications and efficacy, here we will only report studies that used the DDM or a closely related version such as the attentional DDM (Krajbich et al., 2010).

**Figure 3:**
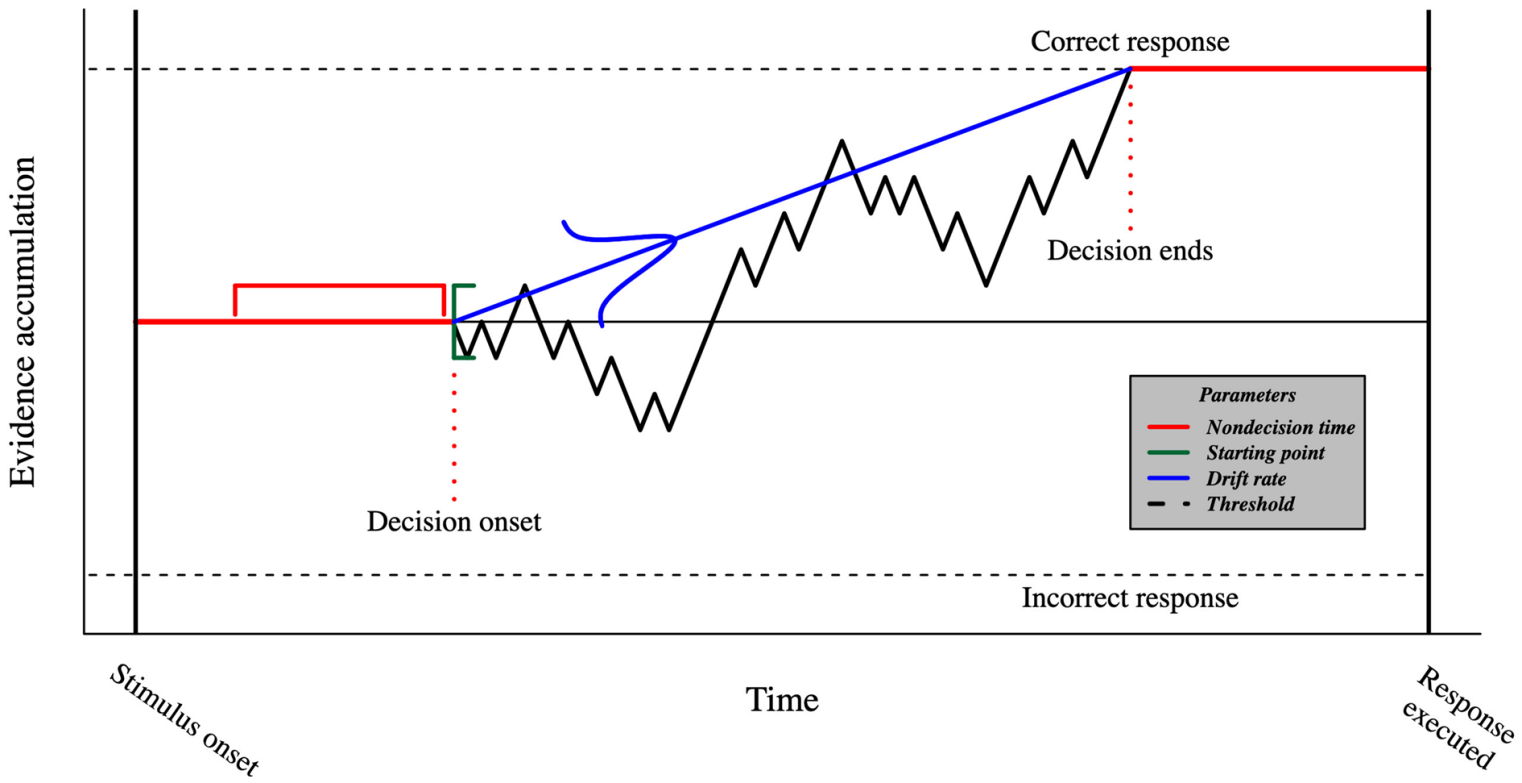
Visual representation of the DDM. The timeframe between stimulus onset and the execution of the response includes both decision- (black) and non-decision- (red) related processes. The evidence accumulation begins at a starting point that is sampled from a uniform distribution (green), and proceeds with a mean speed known as the drift rate, which is sampled from a normal distribution (blue). Once the threshold is reached, we assume that the participant commits to a choice and initiates the motor command. The duration of non-decision processes is sampled from a uniform distribution.

The DDM describes how sensory evidence is accumulated over time (see Figure 3). The average velocity of evidence accumulation is defined by the *drift rate*. The *starting point* of evidence accumulation is drawn from a uniform distribution and, when unbiased, is assumed to be equally distant from each choice on average so that no option has an advantage over the other. The *threshold* is the evidence accumulation boundary. Once it is reached, the participant commits to a choice and executes the motor processes that allow for a response. Finally, *non-decision time* captures all the remaining cognitive processes that happen between the stimulus onset and the decision time, such as visual encoding time and motor response execution. The DDM also has three additional parameters addressing across-trial variabilities in drift rate, starting point, and non-decision time. These parameters allow the DDM to provide accurate predictions of the law-like patterns in the data (Forstmann et al., 2016), especially predicting different RT distributions for correct and incorrect responses (Laming, 1979).

Despite the advantages EAMs offer over simpler cognitive models or statistical tests, a series of criticisms have been raised towards this class of models. Firstly, some authors (Evans and Wagenmakers, 2020) argue that EAMs have reached a limit in the extent to which they can explain decision-making processes. The high similarity between EAMs renders difficult assessing which cognitive dynamics best explain the decision process. A crucial challenge involves assessing how well neural data can be integrated into the EAM framework. Neural data offer greater possibility of discrimination between different theoretical accounts compared to behavioral data. Secondly, some of the models’ assumptions are incompatible with recent empirical findings (Evans and Wagenmakers, 2020). For example, the motor response and the decision process have been shown to be temporally intertwined (Servant et al., 2015, 2016), contradicting the DDM assumption of sequentiality. A future step to address such a problem will consist of replacing random sources of variability with systematic ones. Other limitations include falsifiability issues which could arise with some EAMs (Heathcote et al., 2014; Jones and Dzhafarov, 2014). If the parameters are not properly constrained, the high flexibility of such models could result in an unfalsifiable model (Heathcote et al., 2014).

## 3. Cognitive modelling of attention and prior probability

### 3.1. Attention

A diverse range of attentional tasks, from Posner Cueing to Multiple Object Tracking, is essential for comprehensively probing the intricate nature of attention. Each paradigm targets specific attentional aspects, such as spatial, feature-based, or object-based attention, enabling a more holistic understanding. As attention is a multifaceted phenomenon, employing various tasks ensures a more accurate and nuanced portrayal of its mechanisms and limitations. This broad perspective is crucial for the development of cognitive models of attention, whose primary goal is to encompass the different attentional mechanisms under a common theoretical framework.

**Figure 4:**
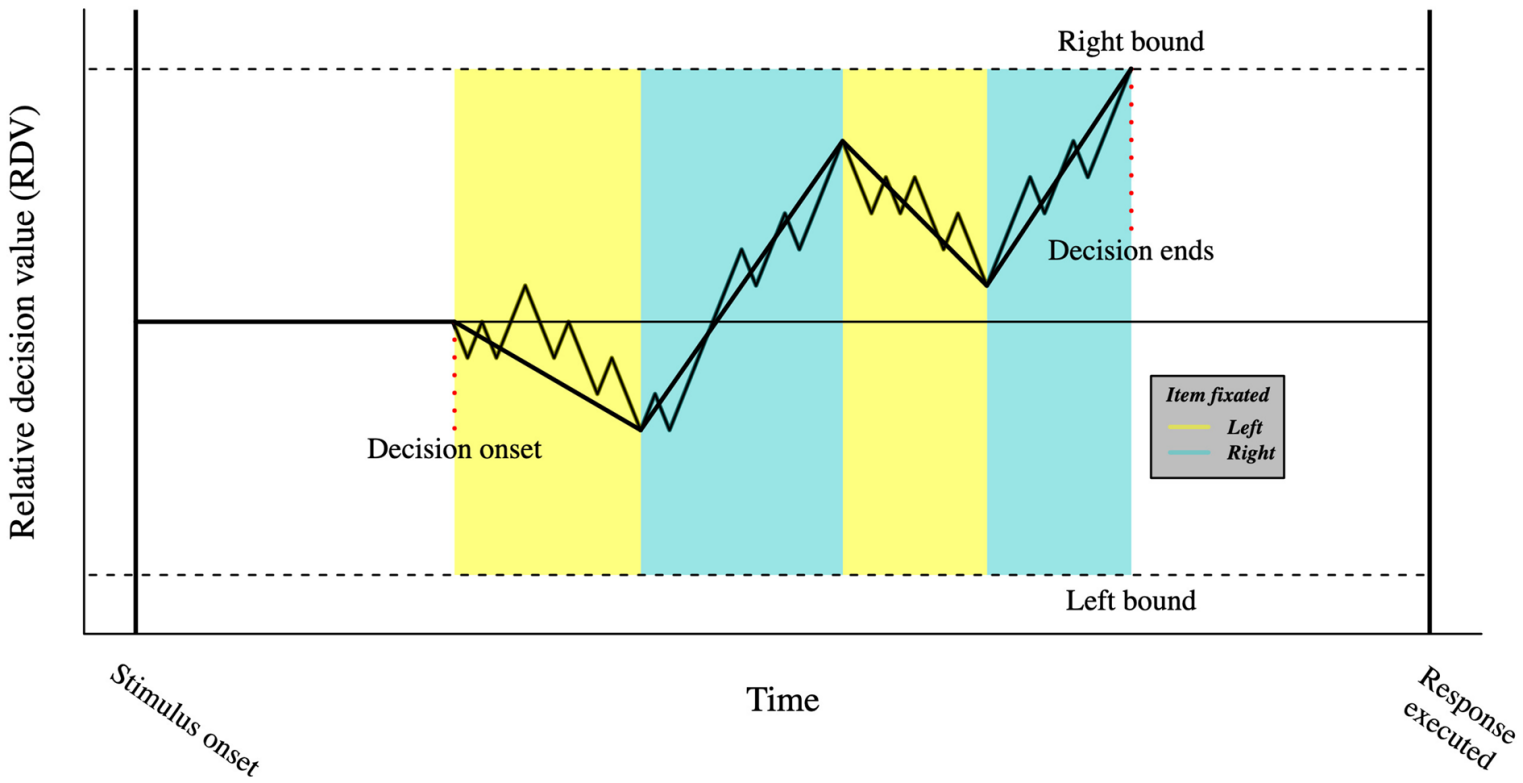
Visual representation of the aDDM. In the aDDM, fixating one option biases the slope of the relative decision value towards that choice. As in the DDM, when the relative decision value reaches one of the boundaries, a decision is made.

An example of this comes from the developed of a variant of the DDM called attentional drift-diffusion model (aDDM; Krajbich et al., 2010). The aDDM is based on the idea that attention mediates the formation of visual short-term memory traces, a concept previously formalized by Smith and Ratcliff (2009). The attentional drift-diffusion model keeps the main properties of the DDM but makes further assumptions about the role of eye movements. Specifically, the model assumes that as participants allocate gaze time onto one option relative to the other, they are more likely to choose the gazed option. Such assumption is formalized as a drift rate bias towards the attended choice (see Figure 4). The aDDM predicts that participants will tend to choose the last seen option, unless the choice’s value is significantly worse than the alternative option; a prediction that has received empirically support (Smith and Krajbich, 2018) across different decision-making domains (e.g., food- and money-related, risky, and social choices), consolidating the role of eye movements in attention orienting. Crucially, promising prospects derive from the recent expansion of the aDDM, in which the probability of fixation on an alternative is expressed as a logistic function of its accumulated value (Gluth et al., 2020). Such models highlight how the attentional mechanisms not only increase evidence accumulation, but also account for the value of the available options (Smith and Krajbich, 2019).

**Figure 5:**
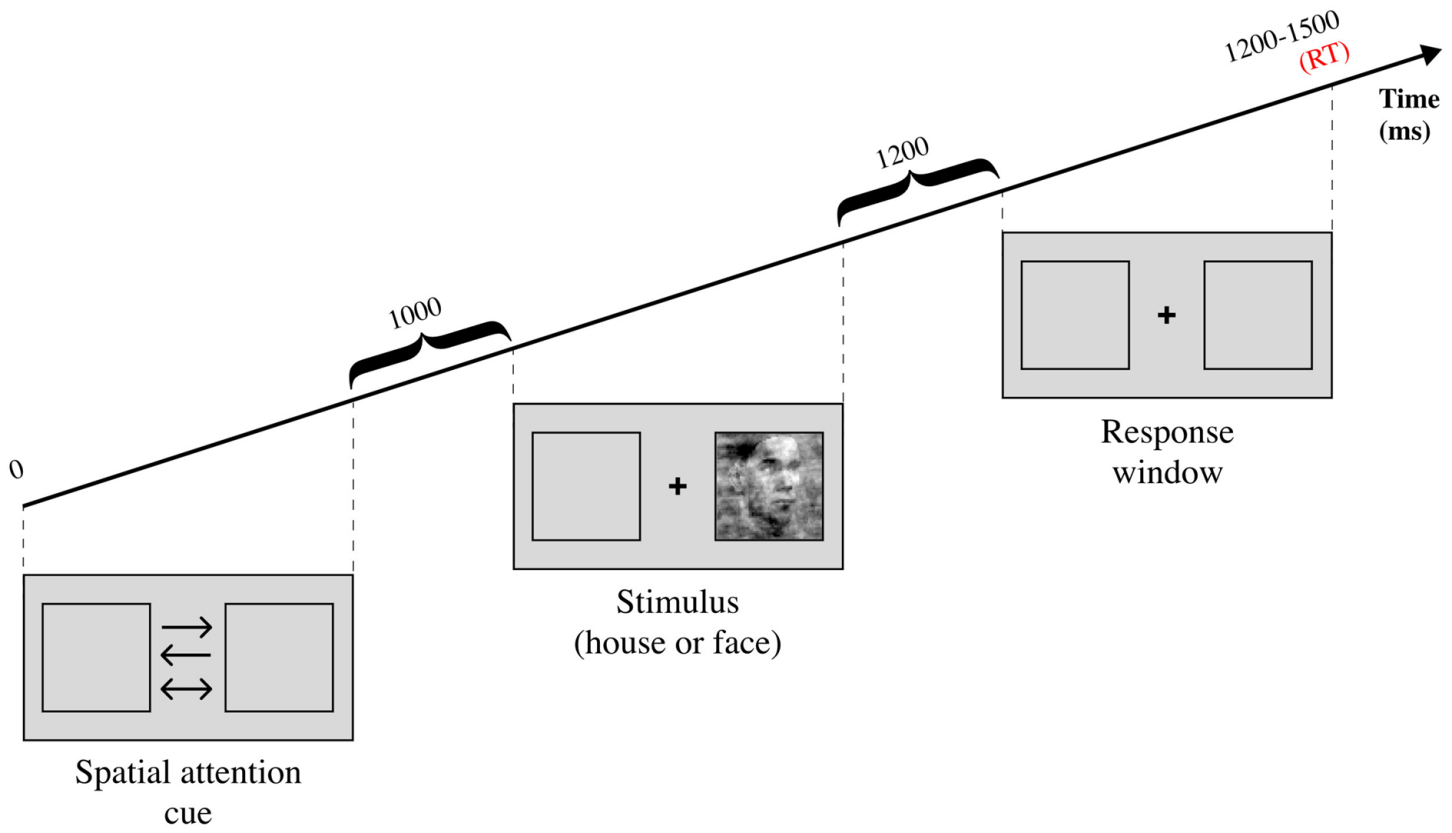
Experimental task from Ghaderi-Kangavari et al. (2022, 2023). There are three possible spatial attention cues, the one-sided arrows are informative while the two-sided arrow is uninformative. The stimulus can either be a face or a house, and it can either have high coherence (low noise) or low coherence (high noise). The participant is asked to report whether the stimulus is a face or a house after the stimulation is over, during the response window.

Two more recent studies investigated how spatial attention influences perceptual decision-making with the classic DDM, while recording electroencephalography (EEG) data. The paradigm, shared between the two studies, consisted in a house/face discrimination task which featured a spatial attention cue before the stimulation (see Figure 5). The first study (Ghaderi-Kangavari et al., 2023) consisted in a model comparison test, measured with deviance information criterion (DIC) and R^2^. The authors reported that the winning model allowed non-decision time to vary between the spatial attention conditions. This finding diverges from the idea that attention affects drift rates by increasing signal-to-noise ratio during evidence accumulation (Smith et al., 2004; Krajbich, 2019; Klatt et al., 2020) but remains in line with DDM predictions (Smith et al., 2004). Nonetheless, this result may not reflect the same attentional processes investigated in previous studies as the uncertainty factor induced by the coherence manipulation influences attentional effects (Smith and Ratcliff, 2009). It is also worth noting that the authors report a significant correlation between the amplitude of contralateral N2 component and non-decision time, corroborating previous studies (Klatt et al., 2020). These findings were extended by the second study (Ghaderi-Kangavari et al., 2022) investigating how spatial attention affects different sub-components of non-decision time. Visual encoding time was found to be influenced by spatial attention but not exclusively. In fact, the authors speculate that motor execution time might be affected as well. Considering that older adults were found to have significantly higher non-decision times compared to younger adults (Klatt et al., 2020), both hypotheses remain plausible.

### 3.2. Prior probability

Several EAM-based studies that probed prior probability manipulations converged on the idea that they mainly influence the starting point parameter (Ratcliff and McKoon, 2008; Simen et al., 2009; Forstmann et al., 2010; Mulder et al., 2012; White and Poldrack, 2014; Garton et al., 2019). More recently, researchers combined the DDM with fMRI data to reveal how prior probability affects neural dynamics during a face/house discrimination task (Dunovan and Wheeler, 2018). The authors reported that the model with the best fit allowed both starting point and drift rate to vary between prior probability conditions. At the neural level, the drift rate bias mapped onto pre- and post-stimuli BOLD activity in the Inferior Temporal Cortex (ITC), indicating that quality of evidence is reflected by physiological activity in this category-selective region. This result corroborates previous findings that found prior probability manipulations to affect the drift rate parameter (Hanks et al., 2011; Kelly et al., 2021). Another recent study (Garton et al., 2019) investigated age-related differences in prior probability bias. The results indicate that flexibility of bias control, which refers to the tendency to be biased, is minimally impaired with age.

### 3.3. Summary

Taken together, studies investigating the role of attention and prior probability via cognitive modelling reveal a complex picture. The discrepancies within studies on attention or expectation might be due to the heterogeneity of paradigms used, deployed strategies by participants or unknown latent factors. Nonetheless, the different impact of age on attention and expectation biases supports the view in which these psychological phenomena arise from different mechanisms.

Attentional manipulations, which have been often linked to the drift rate parameter, and thus to the speed of evidence accumulation, have also been shown to influence non-decision time. Such findings suggest that attention has a broad impact on cognition, which is not measurable by a single DDM parameter. This is in line with the generally shared view of attention as an all-round cognitive phenomenon, not attributable to a single brain region but rather to a network of regions. Components of the frontoparietal network have repeatedly been associated with spatial attention (Nobre and Kastner, 2014), including the posterior parietal cortex, intraparietal sulcus, dorsal premotor/posterior prefrontal cortex and anterior cingulate cortex. On the more temporal side, the results showing a correlation between the contralateral N2 component and non-decision time pave the way for future studies investigating the influence of attention on the motor component of non-decision time, e.g., via electromyography-based models of decision-making (Servant et al., 2021). The overall picture suggests that the selective influence of spatial attention manipulations on DDM parameters is challenged.

Several studies investigating prior probability manipulations have been shown to influence drift rate (Hanks et al., 2011; Dunovan and Wheeler, 2018; Kelly et al., 2021), in addition to the established influence on starting point (Ratcliff and McKoon, 2008; Simen et al., 2009; Forstmann et al., 2010; Mulder et al., 2012; White and Poldrack, 2014; Garton et al., 2019). The diffuse influence of prior probability manipulations on both drift rate and starting point suggests that a multistage process might be in place (Dunovan and Wheeler, 2018). In line with this hypothesis, drift rate between prior probability conditions correlates with the ITC BOLD activity (Dunovan and Wheeler, 2018) while activation in the frontoparietal network is associated with shifts in the starting point parameter (Mulder et al., 2012).

## 4. Conclusion

In this paper, we reviewed the most popular methodological approaches used to study decision-making biases. We illustrated the properties of statistical models and explained why their descriptive function is fundamental to empirical research. Nonetheless, we also discussed how such models do not provide enough insight into the cognitive processes underlying decision-making. We continued by illustrating how cognitive models overcome the limitations of statistical models by providing a formal description of the investigated cognitive processes; the obtained results are unambiguously interpretable according to the model’s assumptions. We reviewed studies that applied both Signal Detection Theory and Evidence Accumulation Models and showed that EAMs have a significant advantage over SDT because they incorporate response time measures, in addition to response choice. Early applications of cognitive models, such as the SDT, provided means to quantify the *bias*. More advanced cognitive models, such as EAMs, can account for the relationship between RT distributions and accuracy (Forstmann et al., 2016). Particularly, the DDM have been shown to reliably explain a variety of speeded decision-making tasks. Finally, we reviewed recent studies which manipulated expectation and attention. Even if the emerging picture remains elusive, cognitive models of decision-making can help us to disentangle intricately intertwined processes such as attention and expectation.

In conclusion, investigating decision-making biases is a necessary step to achieve a greater understanding of the decision-making process. This is true for both basic and clinical research. We argue that, at this point in time, the implementation of state-of-the-art cognitive models is the best approach to quantify biases, test formal theories and advance the field of research.

## 5. Future outlook

While cognitive models are useful for identifying and quantifying (e.g., attentional) biases, they leave open the question of which neural dynamics lead to these biases.

A prominent class of models used to explain the neural dynamics of evidence accumulation are attractor neural network models (Wang, 2002; Wong and Wang, 2006; Quax and Van Gerven, 2018; Esnaola-Acebes et al., 2022). Attractor dynamics entail that the neural networks have one or more stable *states*, to which the network converges over time. These models have also been successful in explaining a wider variety of cognitive processes including memory (Hopfield, 1982) and classification (Chaudhuri and Fiete, 2019). The temporal dynamics of these models can lead to response time and choice data that are highly similar to those predicted by the evidence accumulation models (Keung et al., 2020). Attractor neural network models have been used to investigate which neural parameters lead to known phenomena in RT data. For example, Lo and Wang (2006) used a neural network model of cortico-basal ganglia dynamics to show that adjustments in response caution (decision thresholds) could be implemented by adjusting the strength between synapses of the cortico-striatal connections. Moreover, nonlinear attractor models can explain post-error response time biases in the absence of feedback (Berlemont and Nadal, 2019) and confidence-related sequential effects observed in empirical data (Berlemont et al., 2020).

Despite it is yet unclear how biases in attention or prior probability could be implemented in attractor neural network models, a recent study that fit such models to empirical RT data (Berlemont et al., 2020) offers exciting opportunities for direct comparison with evidence accumulation models.

## Author contributions

EC, SM, and BF contributed to the conception of the article. EC wrote all the sections of the article. SM and BF contributed to manuscript revision and proofreading. All authors contributed to the article and approved the submitted version.
